# Imaging Individual Differences in the Response of the Human Suprachiasmatic Area to Light

**DOI:** 10.3389/fneur.2018.01022

**Published:** 2018-11-29

**Authors:** Elise M. McGlashan, Govinda R. Poudel, Parisa Vidafar, Sean P. A. Drummond, Sean W. Cain

**Affiliations:** ^1^Monash Institute of Cognitive and Clinical Neurosciences and School of Psychological Sciences, Monash University, Melbourne, VIC, Australia; ^2^Sydney Imaging, The University of Sydney, Camperdown, NSW, Australia; ^3^Mary Mackillop Institute of Health Research, Australian Catholic University, Melbourne, VIC, Australia

**Keywords:** melatonin suppression, light sensitivity, circadian rhythms, light exposure, BOLD-fMRI

## Abstract

Circadian disruption is associated with poor health outcomes, including sleep and mood disorders. The suprachiasmatic nucleus (SCN) of the anterior hypothalamus acts as the master biological clock in mammals, regulating circadian rhythms throughout the body. The clock is synchronized to the day/night cycle via retinal light exposure. The BOLD-fMRI response of the human suprachiasmatic area to light has been shown to be greater in the night than in the day, consistent with the known sensitivity of the clock to light at night. Whether the BOLD-fMRI response of the human suprachiasmatic area to light is related to a functional outcome has not been demonstrated. In a pilot study (*n* = 10), we investigated suprachiasmatic area activation in response to light in a 30 s block-paradigm of lights on (100 lux) and lights off (< 1 lux) using the BOLD-fMRI response, compared to each participant's melatonin suppression response to moderate indoor light (100 lux). We found a significant correlation between activation in the suprachiasmatic area in response to light in the scanner and melatonin suppression, with increased melatonin suppression being associated with increased suprachiasmatic area activation in response to the same light level. These preliminary findings are a first step toward using imaging techniques to measure individual differences in circadian light sensitivity, a measure that may have clinical relevance in understanding vulnerability in disorders that are influenced by circadian disruption.

## Introduction

The human circadian system is responsible for regulating physiological processes across the 24-h day. This includes rhythms in alertness, sleep-wake behavior, metabolism, mood and cognitive function (1–3). The endogenous master clock (the suprachiasmatic nucleus, SCN) generates rhythms of ~24 h, which are synchronized to the environmental light/dark cycle via retinal light exposure (4).

Disrupting the relationship between the light-dark cycle, behavior and internal rhythms has significant consequences for health. Circadian disruption is a factor in the etiology of mood disorders (5), cognitive decline (6), the onset of metabolic diseases such as diabetes (3, 7), cardiovascular health (8), and is associated with an increased risk for cancer (9). Although these health concerns may arise from the uncoupling of rhythms with behavior (e.g., cross-meridian travel, engaging in shift-work), it has also been suggested that an abnormal response to environmental light may lead to the development of circadian disruption in the absence of, or in combination with, behavioral change (10, 11). Both hyper- and hypo- sensitivity to environmental light could lead to the development of abnormal circadian synchronization (10–12). Therefore, an abnormal response of the circadian system to light is a potentially important factor for disease vulnerability.

Better characterization of the function of the SCN (master circadian clock) in response to light cues may provide clinically relevant information, leading to improved interventions. However, our understanding of human SCN function in a clinical context to date has often relied on peripheral measurements of clock function. For example, the most common assessments of SCN function involve measuring the timing of melatonin onset (usually via dim-light melatonin onset; DLMO) for circadian timing [e.g., 13, 14], and melatonin suppression to assess circadian light responsiveness [e.g., 11, 15]. However, for patients taking beta-blockers, antidepressants, or sleeping aids such as exogenous melatonin, these assessments will be uninformative due to the pharmacological impact on endogenous melatonin levels, or cross reactivity with existing assays (16, 17). The ability to directly assess the activity of the SCN in response to light cues would overcome these limitations.

There is a substantial neuroimaging literature examining non-visual light responses in humans. For example, the BOLD-fMRI response of the suprachiasmatic area to light during the day, evening, and night has been imaged, showing differential activation across times of day which matches the known rhythm in the responsiveness of the circadian system to light (18). Studies have also shown enhancement of activity in brain areas associated with working memory, alertness and cognition [e.g., 19, 20] and emotional processing (21) in response to blue light, compared to green. Further, the use of light stimuli which differentially stimulate melanopsin (high- or low-stimulation) during fMRI has been utilized to characterize the cerebral activation associated with non-visual light processes (22). However, the measurement of suprachiasmatic area function in humans has yet to be related to individual responsiveness using established laboratory techniques. In this study we examined, within individuals, the relationship between suprachiasmatic area activation in response to light in an fMRI scanner and melatonin suppression to light in the laboratory. We hypothesized increased activation of the suprachiasmatic area in response to light would be associated with increased melatonin suppression to light.

## Materials and methods

### Participants

Ten healthy young men and women (5 men, *M*_*age*_ = 20.80, *SD* = 1.87) were recruited. Participants were free of medical and psychiatric conditions and were not taking any medications at the time of the study. Women were naturally cycling (i.e., not using any hormonal contraception).

### In-laboratory circadian assessments

All participants completed an in-laboratory assessment of circadian light sensitivity. This involved an assessment of dim-light melatonin levels and a subsequent light exposure of ~100 lux. Sessions ran from ~4 h prior to the participants' bedtime, until 1 h after, during which the participant remained awake and seated (other than for bathroom breaks). These two sessions were a minimum of 1 week apart, with the dim-light session occurring first. Participants maintained a strict 8:16 h sleep-wake dark-light schedule for at least 1 week prior to, and in between sessions, whereby >1 deviation of more than 30-min in 1 week would be exclusionary. Adherence to the schedule was monitored using wrist-worn actigraphy (Actiwatch Spectrum Plus or L, Philips Respironics, OR, USA) and sleep diaries. Schedules were selected to be in line with participants typical sleep-wake behavior, an example schedule, with an overview of the protocol is available in the Supplementary Material. During test-sessions, hourly saliva samples were taken using salivettes (Sarstedt, Germany), which were then assayed in duplicate for melatonin at the Adelaide Research Assay Facility using radioimmunoassay with the G280 antibody and the [1251]2-iodomelatonin radioligand (LOD 4.3 pMol).

### In MRI light exposure and imaging procedure

Participants completed an fMRI scan beginning ~1 h prior to habitual bedtime. For 1 h prior to this they were seated in dim-lighting conditions of < 10 lux. Prior to their scan, participants provided a urine sample for toxicology to be conducted, with a positive result being exclusionary (*n* = 0, SureStep 6 Panel, Medvet, South Australia, Australia).

All subjects were imaged using a 3T Scanner (Siemens Magnetom Skyra) with 20 channel head coils. High-resolution anatomical images of the whole brain were acquired using T1-weighted anatomical scans (TE = 2.07 ms; TR = 2.3 s; field of view: 256 × 256 mm; slice thickness: 1 mm). Functional images were acquired using echo-planar-imaging (TR: 2.06 s; TE: 24 ms; field of view: 190 × 190 mm; slice thickness: 3 mm; number of slices: 41; flip angle = 90, number of volumes = 177). The first five images of each session were discarded to allow for T1 equilibration.

Participants were requested to lay supine in the MRI scanner, while an optic-fiber-based light delivery system was fitted on the MRI head coil. This device consisted of a halogen light source (DC950H, Dolan-Jenner Industries, MA, USA), which transmitted light through metal-free fiber optic cables (100 strand cable with 0.75 mm fibers, Optic Fiber Lighting, Sydney, AU) to two circular plastic diffusers (40 mm diameter) positioned ~50 mm above each eye. The diffusers were designed to bathe each eye in light, achieving an even spread of illumination. Light stimuli had a CCT of ~2800 K (λp = 650 nm), and was delivered at two intensities, ~100 lux (42.73 μW/cm^2^) and ~1000 lux (392.28 μW/cm^2^).

Each participant was exposed to a passive light stimulus paradigm in which they were requested to keep their eyes open (other than normal blinking). This was comprised of alternating periods of lights off (darkness, six 30 s epochs) and lights on at a moderate level (100 lux, six 30 s epochs) or bright level (1000 lux, six 30 s epochs). Moderate and bright blocks (of 6 min total duration each) were delivered separately, with the moderate light exposure block always being presented first. Due to the aversive nature of the 1000 lux bright-light stimuli (which often led to significant eye closures), only data for the moderate light exposures are reported here.

### Data analysis

#### Melatonin suppression

Area under the curve (AUC) was calculated for the final 2 h of each dim-light control, and each 100-lux light exposure (where melatonin levels were adequate in all participants in our protocol). Average percent suppression across the 2 h was then calculated by determining the percent change in AUC from baseline to the 100-lux light exposure for each individual.

#### MRI data processing

**Figure 1:**
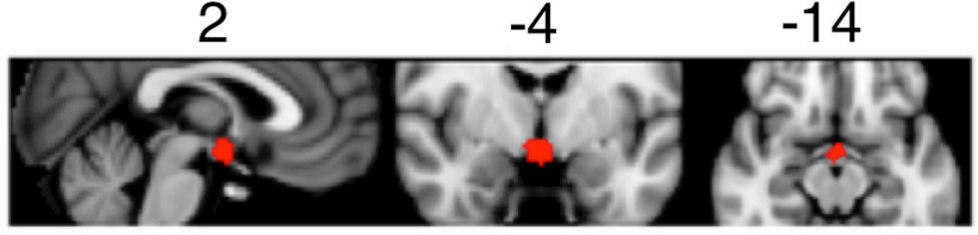
Mask of the suprachiasmatic area used to determine BOLD activation during the light exposure paradigm.

Detailed information regarding fMRI data processing and analysis can be found in the Supplementary Material. Briefly, MRI data were pre-processed using FSL (FMRIB's Software Library, www.fmrib.ox.ac.uk/fsl). For each participant, pre-processed fMRI data were analyzed using first-level general linear models. The linear models included regressors for light on blocks and standard motion parameters (six regressors). To focus our analysis on the suprachiasmatic area of the brain, we generated a mask covering hypothalamic area using a meta-analytic tool NeuroSynth (http://neurosynth.org/analyses/terms/hypothalamus/). This mask (see Figure 1) covered both the anterior and posterior hypothalamus including the suprachiasmatic area.

#### Statistical analyses

A correlational analysis was used to assess the relationship between suprachiasmatic area function in response to light (100 lux relative to dark periods) and melatonin suppression. A Spearman's correlation was used due to the small sample size and potential non-normality of the BOLD response.

## Results

**Figure 2:**
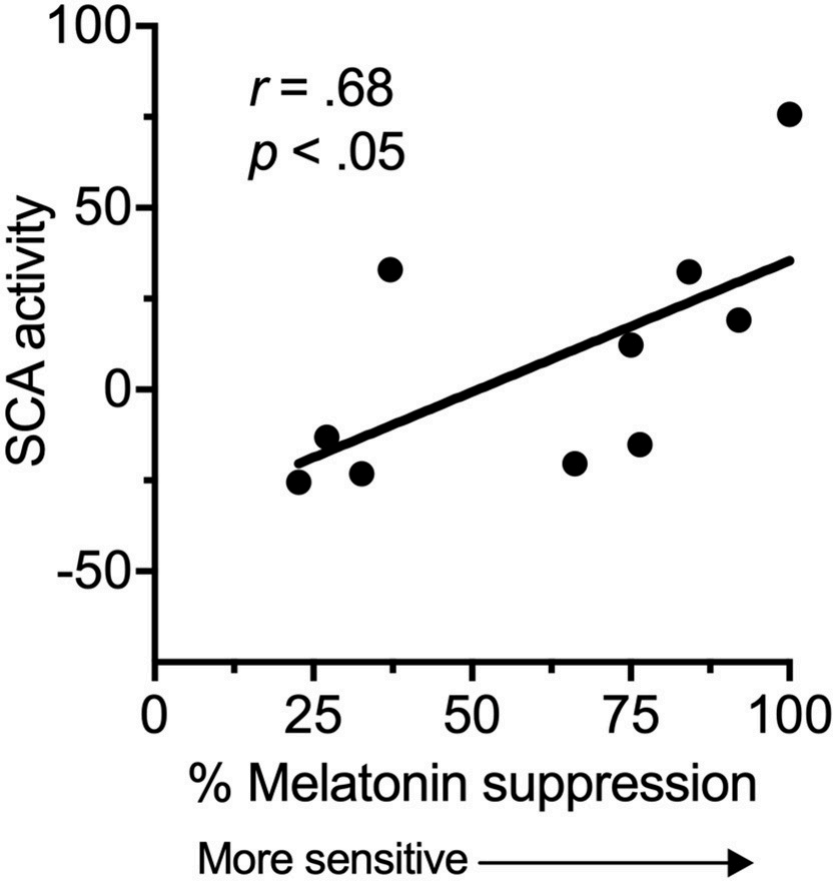
Relationship between the fMRI BOLD response in the suprachiasmatic area (SCA) during 100 lux light exposures (relative to dark), and melatonin suppression.

There was a significant, strong positive correlation between suprachiasmatic area activity during light exposure periods (relative to dark) and melatonin suppression (Figure 2). Increased suprachiasmatic area activation was associated with an increase in melatonin suppression (i.e., greater circadian light sensitivity).

## Discussion

This study provides preliminary evidence for a relationship between suprachiasmatic area activation in response to light and an established in-laboratory measure of circadian light sensitivity. We found a significant relationship between suprachiasmatic area activation and melatonin suppression, indicating that an increase in fMRI measured suprachiasmatic area activation in response to light related to an increase in circadian light sensitivity. Thus, these are the first data in humans to show a relationship between a proximal measure of activity in the anterior hypothalamus and a functional outcome.

An increase in melatonin suppression relates to larger shifts in circadian phase (23), and has been associated with disease states (10, 11). Our results suggest that increased melatonin suppression findings may reflect increased activation of the SCN in response to environmental light. Light information is received at the retina by intrinsically photosensitive retinal ganglion cells (iPRGCs), which then project to the SCN via the retinohypothalamic tract (RHT), and to other brain areas (24, 25). Light exposure leads to changes in circadian timing, amplitude, levels of alertness and mood (23, 26, 27). The magnitude of the impact of this light on the circadian system will be partly dependent on individual differences in light sensitivity, and our results demonstrate that this inter-individual variability may arise from functional differences in the ability of retinal light exposure to activate the SCN.

Circadian dysfunction has been associated with several chronic disease states, including mood disorders (10, 28), metabolic and cardiovascular disease (29) and sleep disorders (11, 14, 30). Abnormalities in circadian light sensitivity may be a trait vulnerability for mood disorders with variable or decreased sensitivity being observed in seasonal affective disorder (12), while hypersensitivity to light has been observed in bipolar disorder (10, 28), and in some sleep disorders or disturbances (11, 31). Imaging of the response to moderate light as used in this study may reveal abnormal SCN function, which could lead to circadian dysfunction.

It should be noted that although a significant relationship was observed here between suprachiasmatic area activation and melatonin suppression, our sample was small, and these data do not indicate that an individual scan of the response to light can currently replace melatonin suppression as an indicator of circadian light sensitivity. The BOLD fMRI response to light in the suprachiasmatic area may instead prove a useful clinical tool for studying changes in light sensitivity associated with either a clinical diagnosis, or pharmacological intervention. Given suggestions that light sensitivity can change across a disease course (12), and may mediate treatment response in mood disorders (32, 33), this has important clinical implications. However, further characterization of the relationship between suprachiasmatic area activation and melatonin suppression is required in order to establish clinically meaningful ways of interpreting individual data.

This study has shown, in a small sample, evidence for a relationship between suprachiasmatic area BOLD-fMRI activation to light and an established measure of circadian light sensitivity. This is a first step in the development of imaging techniques for the assessment of individual differences in circadian function. This is critical given the pervasive nature of circadian dysfunction in disease states.

## Ethics and data availability statement

All procedures were approved by the Monash University Human Research Ethics Committee (MUHREC) prior to commencement (Project 4760). Participants gave written, informed consent prior to participation and were reimbursed for their time. The raw data supporting the conclusions of this manuscript will be made available by the authors, without undue reservation, to any qualified researcher.

## Author contributions

The study was conceived by SC. All authors contributed to the study design. GP was responsible for programing the light delivery device and MRI sequences, and completing fMRI data analysis. EM and PV were responsible for recruitment, data collection, and melatonin data processing. EM was responsible for the final data analysis and writing the manuscript. All authors reviewed and contributed to the manuscript prior to submission for publication.

### Conflict of interest statement

The authors declare that the research was conducted in the absence of any commercial or financial relationships that could be construed as a potential conflict of interest.
