# Supplementary material for: Imaging Individual Differences in the Response of the Human Suprachiasmatic Area to Light
Source: Front Neurol. 2018 Nov 29;9:1022. doi: 10.3389/fneur.2018.01022 (PMC6281828; doi:10.3389/fneur.2018.01022)
Supplement: Supplementary file 1 [file Data_Sheet_1.docx]

Supplementary Material

Imaging individual differences in the response of the human suprachiasmatic area to light

EM McGlashan, G Poudel, P Vidafar, SPA Drummond & SW Cain*

*** Correspondence:** Sean Cain: [sean.cain@monash.edu](mailto:sean.cain@monash.edu)

**1 Supplementary methods and data analysis**

**fMRI data processing and analysis***Pre-processing of MRI data*
The MRI data were pre-processed using FSL (FMRIB's Software Library, [www.fmrib.ox.ac.uk/fsl](http://www.fmrib.ox.ac.uk/fsl)). fMRI pre-processing included motion correction (Jenkinson et al., 2002), slice-time correction, spatial smoothing with a 5-mm Gaussian kernel (full width at half maximum), and high-pass filtering with a cut-off of 120 s. The structural images were registered to the MNI152 standard space using a non-linear registration tool (FNIRT). Registration parameters produced by the non-linear registration process were then used to warp the fMRI images into a standard 2 × 2 × 2 mm^3^ Montreal Neurological Institute (MNI) template. Non-linear registration was used to ensure that small structures are aligned properly.

*fMRI data analysis*For each participant, pre-processed fMRI data were analysed using first-level general linear models. The linear models included boxcar regressors for light on blocks and standard motion parameters (six regressors). Regressors were convolved with double-gamma haemodynamic response function before estimation of first-level model. The parameter estimates of contrast of interest were combined to across subjects for a group-level analysis.

To focus our analysis on Suprachiasmatic area of the brain, we generated a mask covering hypothalamic area using a meta-analytic tool NeuroSynth (<http://neurosynth.org/analyses/terms/hypothalamus/>). This mask (Figure 1) covered both anterior and posterior hypothalamus including the suprachiasmatic area.

Parameter estimates maps were analysed in a group-level analysis. Significance of activity difference between 100 lux and baseline exposure was tested using small volume cluster-level correction (using the hypothalamic mask) and thresholded at *p*-value <0.05 (Fig S1). Parameter estimates from significant regions were extracted for visualisation and correlation purposes.

We further validated the association between melatonin suppression data and BOLD fMRI by running group-level regression analysis between BOLD activity and melatonin suppression data. A group-level regression model included mean and melatonin suppression data as predictors. The relationship was examined at group-level using non-parametric permutation tests (n = 5000 permutations) with small-volume (using the suprachiasmatic area mask) cluster thresholding (p<0.05) using randomise in FSL (Fig S1).

# 2 Supplementary Figures and Tables

## 2.1 Supplementary Figures

1.6 -1.8 -14


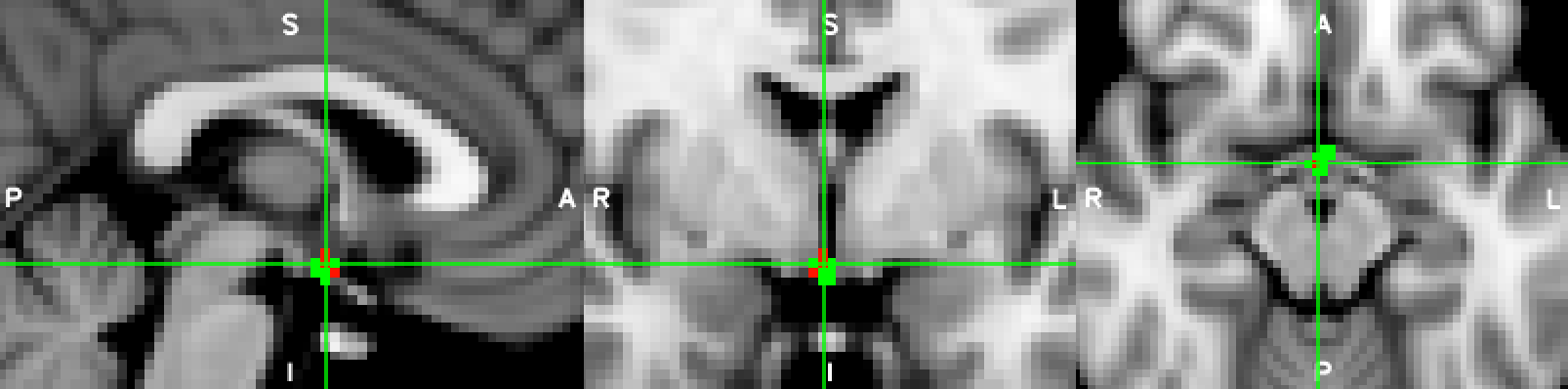


**Supplementary Figure 1.** BOLD activity in SCA during 100 lux light exposure. Significant activity (p<0.05, small volume correction) was observed within SCA (green voxels) during light exposure. There was a significant correlation (p<0.05, small volume correction) between BOLD activity during light exposure and melatonin suppression.


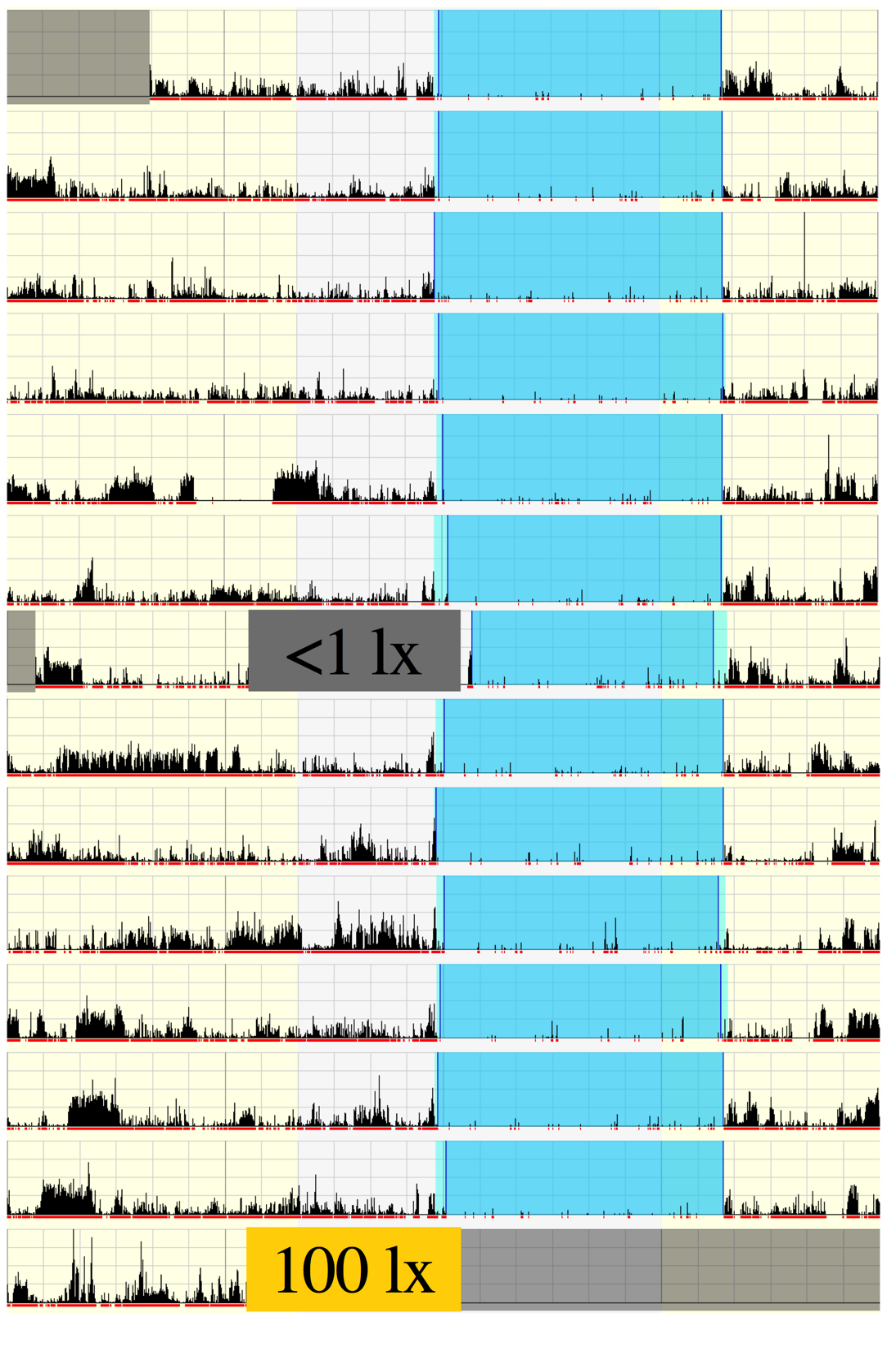


**Supplementary Figure 2.** An overview of the melatonin suppression protocol, with an example sleep-wake schedule for one subject. Blue blocks indicate automatically scored sleep periods (Actiware, Phillips Respironics, OR, USA).
